# Have We Reached a Steady State Between Older Male and Female Labor Force Participation Rates?

**DOI:** 10.1093/geroni/igaf122.1435

**Published:** 2025-12-31

**Authors:** Kevin Cahill, Michael Giandrea, Joseph Quinn

**Affiliations:** FTI Consulting, Inc., Boise, Idaho, United States; U.S. Bureau of Labor Statistics, Washington, District of Columbia, United States; Boston College, Chestnut Hill, Massachusetts, United States

## Abstract

Older workers’ labor force participation rates have stabilized in recent years, after increasing for more than two decades. This apparent change in trend contrasts with one among younger workers, who have experienced increases in labor force participation in the wake of the Covid-19 pandemic. A narrative that older workers are the exception to reductions in labor force participation generally may be coming to an end. This paper explores changes in older workers’ labor force participation rates, with a focus on gender differences, to assess whether gender differences have also stabilized in recent years. We find that a change in trend occurs among older women around 2010, with a less prominent one occurring among older men around the same time. Year-on-year increases in older women’s labor force participation rates are now more or less aligned with those of older men, the product of post-World War II trends, with large numbers of women entering the labor force, having run their course. Policymakers interested in promoting continued work later in life among older workers may need to reflect on gender differences in new ways, at least with respect to overall trends.

